# Longitudinal analysis of vertebral fracture and BMD in a Canadian cohort of adult cystic fibrosis patients

**DOI:** 10.1186/1471-2474-9-125

**Published:** 2008-09-19

**Authors:** Alexandra Papaioannou, Courtney C Kennedy, Andreas Freitag, John O'Neill, Margaret Pui, George Ioannidis, Colin Webber, Anjali Pathak, Suzanne Hansen, Rosamund Hennessey, Jonathan D Adachi

**Affiliations:** 1 Department of Medicine, McMaster University, 1200 Main St W., Hamilton ON, L8N 3Z5, Canada; 2 Hamilton Health Sciences, McMaster University Site, 1200 Main St W., Hamilton, ON, L8N 3Z5, Canada; 3 St. Joseph's Healthcare/McMaster University, 1200 Main St W., Hamilton ON, L8N 3Z5, Canada; 4 Department of Diagnostic Imaging, Scarborough Hospital, General Division, 3050 Lawrence Avenue E., Scarborough, ON, M1P 2V5, Canada; 5 Department of Nuclear Medicine, McMaster University, 1200 Main St W., Hamilton ON, L8N 3Z5, Canada

## Abstract

**Background:**

Vertebral fractures in patients with cystic fibrosis (CF) may contribute to an accelerated decline in lung function and can be a contraindication to lung transplantation. In this study, we examined longitudinal change in bone mineral density (BMD) and the prevalence of vertebral fractures in adult CF patients, without lung-transplant, attending a Canadian specialty clinic.

**Methods:**

Retrospective chart review of all patients attending an Adult Cystic Fibrosis Clinic at Hamilton Health Sciences in Hamilton, Canada. Forty-nine of 56 adults met inclusion criteria. Chest radiographs were graded by consensus approach using Genant's semi-quantitative method to identify and grade fractured vertebrae. Dual x-ray absorptiometry (DXA) scans were also reviewed.

**Results:**

The mean age of the cohort was 25.2 years (SD 9.4), 43% were male. The mean body mass index (BMI) was 19.8 (2.8) for males and 21.7 (5.1) for females. At baseline, the rate of at least one vertebral fracture was 16.3%; rising to 21.3% (prevalent and incident) after a 3-year follow-up. The mean BMD T-or Z-scores at baseline were -0.80 (SD 1.1) at the lumbar spine, -0.57 (SD 0.97) at the proximal femur, and -0.71 (SD 1.1) at the whole body. Over approximately 4-years, the mean percent change in BMD was -1.93% at the proximal femur and -0.73% at the lumbar spine.

**Conclusion:**

Approximately one in five CF patients demonstrated at least one or more vertebral fractures. Moderate declines in BMD were observed. Given the high rate of vertebral fractures noted in this cohort of adult CF patients, and the negative impact they have on compromised lung functioning, regular screening for vertebral fractures should be considered on routine chest radiographs.

## Background

Cystic Fibrosis (CF) is among the most common fatal autosomal recessive genetic diseases in the Caucasian population. Since those born with CF are now living significantly longer, there is a definite need to address a range of erging comorbid conditions, which were not previously considered for a CF population. Low bone mineral density (BMD) is common in adult CF patients and has been termed CF-related bone disease [1,2]. The underlying etiology of low BMD is likely multi-factorial [1-4], however several reports support the hypothesis that the inflammatory response to chronic pulmonary infection plays a significant role in bone loss [1,3,5].

In non-CF populations, vertebral fractures are the most common type of osteoporotic fracture [6] and have important consequences, including increased risk for subsequent fracture [7-11], increased mortality [8,12-15], and reduced quality of life [16-19]. In a CF population, a further consequence of a vertebral (thoracic) fracture is an accelerated decline in lung function (i.e. structural alteration of the chest wall) and contraindication to lung transplantation, an important treatment option for CF patients [1,2]. As CF patients often have routine chest radiographs, screening for vertebral fracture may be an important consideration in this population.

The pathophysiology of CF-related bone disease is not well understood and there are few longitudinal reports on BMD and vertebral fracture. Previous studies in this area have examined CF patients cross-sectionally or only over a short term. Two recent cross-sectional studies found a high rate of vertebral fracture in general CF populations [20,21].

In our Canadian cohort, the purpose of this study was to examine: 1) longitudinal changes in BMD and 2) the rate of vertebral fractures in adults with CF, who have moderate to severe respiratory impairment and who have not undergone lung-transplant.

## Methods

### Study population

All patients who attended the Adult Cystic Fibrosis Clinic at Hamilton Health Sciences, McMaster University Medical Centre (Hamilton, Canada) during 2002 were considered for this study. To be included, patients must have had at least one chest radiograph or DXA scan in previous years (completed as part of routine care in the CF clinic). Patients who were accepted on a lung-transplant list or had received a prior organ transplant were excluded. CF was confirmed by a positive sweat test and deoxyribonucleic acid (DNA) analysis. Of 56 individuals attending the clinic during 2002, four were excluded due to the unavailability of radiographic results, and three patients were excluded due to a past lung transplant. The final cohort consisted of 49 patients. The Institutional Review Board at McMaster University approved the study.

### Radiology review

The first and last available routine chest radiographs (separated by at least one year) taken between May 1996 and December 2003 were selected for each participant. Two board certified radiologists (authors MP and JO) independently reviewed chest radiographs (lateral and anterior posterior views) using a modified Genant's semi-quantitative method [22,23]. This method distinguishes fractured vertebrae (grades 1, 2 and 3) from non-fractured vertebrae (grades 0 for no fracture, 0.5 for reduction of height of less than 20% or reduction in area of less than 10%). A deformity graded 1 and higher (excluding congenital or degenerative causes) indicates a reduction in vertebral height (anterior, posterior, or middle) of greater than or equal to 20–25%. Grade 1 is considered a mild fracture, Grade 2 a moderate fracture, and Grade 3 a severe fracture. Differences of scores between radiologists were resolved by consensus.

### Bone densitometry

A medical physicist (author CW) reviewed all annual DXA scans of the lumbar spine, proximal femur (total region), and whole body taken during the study period. All DXA scans were performed on a Hologic QDR 4500A densitometer (Hologic Inc., Bedford, MA, USA). BMD measurements were reported in g/cm^2^, and T- or Z-scores. Expression of an adult BMD as a T-score incorporates the typical BMD loss from peak bone mass that is associated with aging as well as any BMD loss relative to an age-matched peer. By definition, a T-score is not applicable to children. Consequently for subjects aged 19 years or less, BMD values were expressed as Z-scores.

### Clinical/laboratory variables

Research assistants abstracted clinical and demographic data from clinic charts for the nearest clinic visit within 9-months of both the baseline radiograph and DXA scan (i.e. if taken at non-coincident time periods). Data abstracted included height, weight; use of inhaled steroids, bisphosphonates, hormone replacement therapy, oral contraception, calcium and vitamin D supplements; laboratory tests and pulmonary functioning including forced expiratory volume in 1 second (FEV^1^) and forced vital capacity (FVC). Dietary calcium intake was not available. Charts were also reviewed for any evidence of oral corticosteroid use between the baseline and follow-up radiograph/DXA examinations and the cumulative number of milligrams taken was recorded.

Routine laboratory results were downloaded from the computerized hospital database. Phosphate, alkaline phosphatase, serum calcium, albumin, alanine aminotransferase (ALT), g-glutamyltransferase (GGT) and creatinine were measured with automatic analyzers. Commercially available kits (Nichols Institute Diagnostics, San Clemente, CA, USA) were used to measure parathyroid hormone (PTH; Nichols Advantage Bio-Intact PTH (1–84) immunometric assay) and 25-hydroxyvitamin D (25-OHD; Nichols Advantage 25-Hydroxyvitamin D assay).

### Statistical analysis

Analyses were performed with SPSS (version 13.0; SPSS Inc., Chicago, IL, USA) and SAS/STAT (version 8.0; SAS Institute Inc., Cary, NC, USA) software packages. Clinical and laboratory variables are summarized as means and standard deviations, and medication/supplementation use (yes/no) as proportions. Due to deviations from normality and the small sample size of the fracture group, differences in baseline characteristics (continuous variables) between fracture versus non-fracture patients were determined by Mann-Whitney U-test. Fisher's exact test was used to examine differences between proportions.

For the BMD analysis, all individuals had two to five BMD measures during the course of the study. To incorporate all BMD measurements, a regression slope was calculated using all available measurements for each patient. The slope represents the average yearly bone mineral density change over the entire follow-up period for each patient. This analysis takes into account the differing numbers of BMD measurements and the length of follow-up for each patient. Separate analyses were performed for the lumbar spine, whole body, and proximal femur (average of right and left sides). Baseline BMD and variables that were significant in univariate analysis were included in multi-variable regression models to estimate percent BMD change per year. In all analyses, a 2-sided p-value of < 0.05 was considered significant.

## Results

Overall, the mean age of the cohort was 25.2 years (SD 9.4), 43% were male. Seventy-five percent of the sample was between ages 15–31 years; 25% were ages 32–51 years. The median age was 23.0 years. Table 1 displays baseline patient characteristics for the fracture (prevalent/baseline) and non-fracture groups. The mean age of fracture patients was significantly higher than that of non-fracture patients (32.7 years versus 23.8). Males with vertebral fracture had significantly higher weight and body mass index (BMI) scores than those without. Several of the laboratory variables were higher in the fracture than non-fracture group (Table 1), however we lacked power to see this statistically. Creatinine was significantly higher in the fracture group.

**Table 1:** Baseline characteristics* for the entire study cohort and by vertebral fracture status

		Overall n = 49	Nonfracture Group n = 41	Fracture Group^a ^ n = 8	p-value
	Valid n, Nonfracture/Fracture				

Male gender, %	41/8	42.9	39.0	62.5	0.263
Age, years	41/8	25.2 (9.4)	23.8 (8.7)	32.7 (10.3)	0.024^†^
Weight, kg					
males	16/5	59.5 (9.2)	56.1 (7.4)	70.4 (5.3)	0.003^†^
females	25/3	57.4 (16.4)	57.3 (17.5)	58.0 (11.5)	0.726
Height, cm,	41/8	166.8 (7.9)	166.1 (7.7)	170.3 (8.9)	0.175
BMI, kg/m^2^					
males	16/5	19.8 (2.8)	18.9 (2.2)	22.8 (2.4)	0.004^†^
females	25/3	21.7 (5.1)	21.7 (5.3)	22.3 (3.7)	0.726
FEV_1_	39/8	2.2 (0.9)	2.2 (0.9)	2.5 (0.7)	0.202
FVC	39/8	3.1 (0.9)	3.0 (0.9)	3.4 (0.9)	0.098
Hemoglobin g/L	38/7	139.2 (14.8)	138.1 (14.6)	144.9 (15.2)	0.301
Creatinine, umol/L	34/7	66.8 (17.4)	64.8 (18.1)	76.0 (10.0)	0.045^†^
Calcium, mmol/L	34/5	2.3 (0.1)	2.3 (0.1)	2.3 (0.1)	0.657
Albumin, g/L	37/8	39.7 (3.7)	39.8 (3.9)	39.3 (2.5)	0.550
25-hydroxyvitamin D, nmol/L	26/4	61.9 (28.8)	61.5 (31.0)	64.6 (5.4)	0.617
Parathyroid hormone, pg/ml	17/4	28.0 (17.7)	24.8 (18.0)	41.5 (6.7)	0.120
Phosphate, mmol/L	32/5	1.2 (0.2)	1.2 (0.2)	1.3 (0.1)	0.813
Alkaline Phosphatase, U/L	37/8	121.8 (91.4)	116.6 (89.7)	146.1 (101.5)	0.082
Alanine Aminotransferase, IU/L	36/8	32.5 (29.1)	29.6 (22.0)	45.6 (50.5)	0.560
g-glutamyltransferase, U/L	37/7	31.2 (51.8)	26.7 (39.9)	55.1 (94.2)	0.297
Inhaled Steroids, % yes	41/8	55.1	51.2	75.0	0.269
Vitamin D (ADEK), % yes	41/8	55.1	53.7	62.5a	0.715

Data expressed as mean (SD) or proportion.

*Data was abstracted within 9-months of baseline radiograph.

^†^p < 0.05

^a^Fractures present at baseline (prevalent fractures)

BMI, body mass index; FEV_1_, Forced Expiratory Volume in 1 second; FVC, Forced Vital Capacity

At baseline, eight out of 49 patients (16.3%) had at least one prevalent Grade 1 fracture (Table 2). Multiple vertebral fractures were noted in four (8.2%) patients. A second radiograph was available for 47/49 patients. Neither of the two patients without a follow-up radiograph had a vertebral fracture noted at baseline. The mean follow-up between each patient's first and last radiograph was 3.3 years (SD 1.4). During this period, four new fractures occurred (incidence rate of 8.5%); three patients had a new first fracture, and one patient had an additional vertebral fracture since baseline (Table 2). The mean age at follow-up for patients with a new fracture was 35.6 years (SD 14.7) versus 28.1 years (SD 8.8) for patients with no fracture (not statistically significant but lacked power). One patient had a fracture at baseline that was not apparent at follow-up (two grade 0.5 fractures were noted instead of one grade 1 and one grade 0.5 as at baseline). Thus, at follow-up, the overall rate of at least one Grade 1 vertebral fracture (both incident and prevalent) increased to 21.3% of patients (10/47; Table 2).

**Table 2:** Vertebral fractures in the study cohort*

	Baseline n = 49			Follow-up n = 47^†^		
	No. of patients with fracture (at least one)	No. of patients without	Total No. fractures	No. of patients with fracture (at least one)	No. of patients without	Total No. fractures

New Vertebral Fracture (≥Grade 1)	-	-	-	4^a^	43	4
All Vertebral Fracture^b ^(≥Grade 1)	8	41	12	10	37^c^	15

*Mean length of follow-up between baseline and follow-up radiographs was 3.3 years (SD 1.4)

^†^Of the two patients missing a follow-up radiograph, neither patient had a fracture present at baseline.

^a^One patient also had a prevalent fracture at baseline.

^b^For follow-up, this includes both prevalent and incident fractures.

^c^One patient had a fracture indicated at baseline but not on the follow-up radiograph.

During the study period, 14 of 47 (30%) patients had taken oral corticosteroids. The mean cumulative dose per patient was 1517 mg and the mean cumulative number of days taking oral corticosteroids was 37 (SD 21.3). One of these patients taking corticosteroids had a new incident fracture (cumulative corticosteroid dose of 3000 mg over 68 days) and the remainder had no prevalent or incident fractures. Three patients were taking a bisphosphonate; none had a prevalent or incident fracture. The rate of inhaled steroid use and Vitamin D supplementation at baseline was higher in the fracture group, however we likely lacked power to see a statistical difference (Table 1). Only two patients were taking calcium supplementation at baseline, although patients may have been taking a multivitamin and dietary calcium levels were monitored by the clinic nutritionist (results not available).

### Bone mineral density

Table 3 presents mean BMD values in g/cm^2 ^and T-or Z-scores (lumbar spine, proximal femur, and whole body sites) for all DXA measures during the study period (up to five for some patients). The mean BMD T-or Z-scores at baseline were -0.80 (SD 1.1) at the lumbar spine, -0.57 (SD 0.97) at the proximal femur, and -0.71 (SD 1.1) at the whole body. During the study period, 35 of 49 patients had at least two DXA scans; for these patients, the mean BMD percent change/year (adjusted) is presented in Table 4. Over a mean follow-up of 4.03 years (SD 1.45), the overall rate of bone loss was -0.73% at the lumbar spine, -1.93% at the proximal femur, and -0.40% at the whole body. One outlier who had a BMD change of +12%/year was left out of the lumbar spine analysis (the result was attributed to growth stage and a significant increase in BMI).

**Table 3:** Bone mineral density (BMD) over study period*, mean (SD)

	BMD 1 (Baseline)	BMD 2	BMD 3	BMD 4	BMD 5
Lumbar Spine, n	n = 45	n = 36	n = 28	n = 17	n = 10
T-score/Z-score^† ^(SD)	-0.80 (1.10)	-0.91 (1.09)	-0.95 (0.94)	-1.08 (1.01)	-1.17 (1.26)
BMD, g/cm^2 ^(SD)	0.972 (0.12)	0.968 (0.12)	0.968 (0.11)	0.956 (0.11)	0.944 (1.14)
Proximal Femur^a^, n	n = 41	n = 37	n = 29	n = 18	n = 10
T-score/Z-score^† ^(SD)	-0.57 (0.97)	-0.59 (1.02)	-0.71 (0.88)	-0.82 (0.89)	-0.92 (1.14)
BMD, g/cm^2 ^(SD)	0.902 (0.13)	0.898 (0.14)	0.888 (0.12)	0.869 (0.12)	0.857 (0.16)
Whole Body, n	n = 46	n = 36	n = 27	n = 18	n = 10
T-score/Z-score^† ^(SD)	-0.71 (1.11)	-0.50 (1.07)	-0.61 (1.13)	-0.65 (1.04)	-0.72 (1.25)
BMD, g/cm^2 ^(SD)	1.120 (0.97)	1.136 (0.11)	1.123 (0.11)	1.111 (0.10)	1.100 (0.11)

* The mean follow-up time between first and last BMD examination was 4.03 years (SD 1.45)

^†^T-scores for participants over 19 years of age, and Z-scores for those under 19 years of age.

^a^Average of left and right sides

**Table 4:** Multivariable-adjusted percent BMD change/year, mean (SD)

	% BMD change/year (adjusted)
Lumbar Spine	-0.18^a ^(1.59)*
	n = 30
Proximal Femur†	-0.48^b ^(1.39)
	n = 28
Whole Body	-0.10^c ^(1.07)
	n = 35

All available DXA scans per individual were used in calculating the slope of percent BMD change.

*Outlier removed

† Average of left and right sides

^a^Adjusted for baseline BMD; alkaline phosphatase and serum calcium laboratory levels

^b^Adjusted for Forced Expiratory Volume, serum calcium, and dosage of oral steroids

^c^Adjusted for years of follow-up and age

Although we did not have adequate sample size to make a meaningful comparison, we examined the longitudinal BMD change for patients who had a final DXA scan that was within one year of the follow-up chest radiograph (over 80% were performed within the same month). At the whole body, the crude mean percent BMD change over a mean follow-up of 4.1 years was -3.62% (SD 3.46) for fracture patients (incident or prevalent; N = 5), and -1.90% (SD 3.34) for non-fracture patients (N = 22). Correspondingly, at the lumber spine it was -3.69% (SD 8.21) for fracture patients (N = 5) and -1.01% (SD 4.59) for non-fracture patients (N = 23). At the proximal femur (average of right and left), it was -6.04 (SD 12.1) for fracture patients (N = 5) and -2.32 (SD 7.68) for non-fracture patients (N = 23).

## Discussion

This study demonstrates that the prevalence of vertebral fracture in young adults (mean age 25.2, SD 9.4) with CF and without lung transplantation is similar to Canadian population-based rates of vertebral fracture in men and women over age 50 [24]. In the Canadian Multicentre Osteoporosis Study (CaMos), vertebral fracture rates in Canadians over age 50 were 21.5% for men and 23.5% for women. In our CF cohort, the overall rate of at least one vertebral fracture (prevalent and incident) at follow-up was 21.3% and multiple fractures occurred in four of these patients. The incidence of vertebral fractures over a mean follow-up of 3.3 years was 8.5% (10/47). Considering that the patients in our study were on average 25 plus years younger than CaMos participants, and that the CaMos study uses quantitative morphometry which may yield a higher estimate than the semi-quantitative approach (approximately double by one estimate) [25], the rate of vertebral fractures in adults with CF appears high.

Patients with vertebral fracture were significantly older than their non-fracture counterparts (Table 1). Males with vertebral fractures had a greater BMI than those without fractures, although the mean BMI of both groups were in the healthy range.

Previous cross-sectional studies in CF patients with varied disease severity have found vertebral fracture rates of 17% to 26% [26,27]. In a cohort of late-stage cystic fibrosis patients referred for lung transplantation, a review of chest radiographs revealed that 51% of patients had one or more unreported vertebral compression fractures (>20% anterior wedging), and 15% of patients had unreported rib fractures [28,29].

Two recent cross-sectional studies of CF cohorts similar to our study (i.e. general, non-transplant), have also examined vertebral fractures in relation to BMD. A Canadian study [21] and Italian study [20] found a 7% and 27% vertebral fracture rate, respectively. In the former study, cross-sectional BMD was not related to fracture prevalence, and in the latter, cross-sectional BMD was actually higher in the fracture group. Although we were not able to adequately examine this issue due to small sample size, our data suggests that during the study period the crude rate of BMD *loss *was greater in the fracture than non-fracture group. Whether BMD *loss *(versus cross-sectional BMD) is predictive of fracture should be evaluated in a future longitudinal study.

In the general population, once peak bone mass has been reached, the rate of bone loss tends to be somewhat stable with age until the fifth and sixth decades [30]. In our cohort of young adults, we found moderate declines (i.e. -0.73, -1.93, -0.40 percent BMD change, adjusted) in bone loss at the lumbar spine, proximal hip, and whole body in both fracture and non-fracture patients over 4-years of follow-up. Our results exhibit a similar trend to Haworth et al. [31] who examined percent change in BMD over a 1-year follow-up. In that study, annual bone loss occurred particularly for adults >25 years (N = 57), where a decline of -1.9% and -1.5% occurred at the femoral neck and total hip (no loss was observed at the lumbar spine).

Bone quality as opposed to simply quantity or mass, and its relationship to fractures, requires further examination in CF patients. Increasingly, an emphasis on bone quality is emerging in osteoporosis research [32], and this may prove to be a particularly relevant topic to CF patients. BMD as measured by DXA may not adequately predict fracture risk in CF patients [20,21]. Quantitative ultrasound (QUS) is another alternative that provides information on the structural organization of bone in addition to bone mass. QUS may be particularly applicable for patients with reduced bone quality such as patients with corticosteroid induced osteoporosis [33]. In a population-based, epidemiological study (men and women age 42–82 years), QUS of the calcaneum has also been shown to predict fracture risk [34].

Recently, Rossini et al. [35] examined whether QUS could discriminate between adult CF patients with and without vertebral fractures. In the cohort of 172 CF patients, mean age of 27 years, 44% were previously or currently taking oral corticosteroids. Approximately 1 in 3 patients had a vertebral deformity. Overall, only phalangeal QUS as opposed to calcaneal QUS or DXA measures were able to discriminate between patients with and without vertebral fractures. Thus, the hypothesis that CF patients have qualitative alterations in bone, which may be independent of BMD, appears warranted and requires further study.

Our study is limited by a number of factors. We relied on retrospective clinic data extending over a number of years. DXA scans and chest radiographs were considered coincident provided they were performed within one year of each other (however over 80% were within 1-month). The ages of the subjects in our study ranged from 15 to 51 years, with a median age of 23. Thus, both young and middle-aged adults were included, whose clinical and bone-related characteristics may differ.

One patient in our study had a grade 1 fracture indicated at baseline but not on the follow-up radiograph (instead it was graded 0.5). This is a potential problem of Genant's semi-quantitative method used in this study [22,23]. Although this method is probably the most widely cited in osteoporosis studies, it depends on the recognition of the radiological signs of fracture by experienced observers [25]. There is considerable debate regarding the level of deformity and accuracy of reporting, particularly that with Genant's method the separation of the grades is not explicit [25]. Grade 1 is considered a fracture as per Genant's method; grade 0. 5 is an observable deformity but represents a reduction in height or area less than the criterion for fracture.

## Conclusion

Recent studies have examined bisphosphonates for the treatment of low bone mass in CF patients, demonstrating a substantial improvement in BMD after one year [36,37]. Future studies should examine whether bisphosphonate or other treatment in CF patients also produces a consequent reduction in vertebral (and non-vertebral) fractures. Given the high rate of vertebral fractures noted in CF patients, and the potential negative impact they have on compromised lung functioning, regular screening for vertebral fractures should be considered on routine chest radiographs.

## Competing interests

Alexandra Papaioannou:

Consulting Role: Amgen, Eli Lilly, Merck Frosst, Novartis, Proctor & Gamble, sanofi aventis

Clinical Trials: Eli Lilly, Merck, Novartis, Proctor & Gamble, sanofi-aventis

Jonathan D. Adachi:

Consulting Role: Amgen; Astra Zeneca, Eli Lilly; GlaxoSmithKline; Merck Frosst; Novartis; Proctor & Gamble; Roche; Sanofi Aventis; Servier

Clinical Trials: Eli Lilly; GlaxoSmithKline; Merck; Novartis; Pfizer; Proctor & Gamble; sanofi-aventis; Servier; Wyeth-Ayerst

Courtney Kennedy: none to declare

Andreas Freitag: none to declare

John O'Neill: none to declare

Margaret Pui: none to declare

Colin Webber: none to declare

Anjali Pathak: none to declare

Suzanne Hansen: none to declare

Rosamund Hennessey: none to declare

George Ioannidis: none to declare

## Authors' contributions

AP and CK contributed to concept and design of study, analysis and interpretation; and preparation of the manuscript. AF, GI, JA, CW, JO, and MP contributed to concept and design of study, analysis and interpretation; and editing the final manuscript. AP, SH, and RH contributed to concept and design of study, patient recruitment, data collection, and editing the final manuscript. All authors gave final approval to the article submitted.

## Pre-publication history

The pre-publication history for this paper can be accessed here:


